# Quality assurance in 3D-printing: A dimensional accuracy study of patient-specific 3D-printed vascular anatomical models

**DOI:** 10.3389/fmedt.2023.1097850

**Published:** 2023-02-07

**Authors:** Philip Nguyen, Ivan Stanislaus, Clover McGahon, Krishna Pattabathula, Samuel Bryant, Nigel Pinto, Jason Jenkins, Christoph Meinert

**Affiliations:** ^1^ School of Medicine, The University of Queensland, Brisbane, QLD, Australia; ^2^ Faculty of Engineering, Queensland University of Technology, Brisbane, QLD, Australia; ^3^ Vascular Surgery Department, Royal Brisbane and Women's Hospital, Metro North Hospital and Health Services, Brisbane, QLD, Australia; ^4^ Vascular Biofabrication Program, Herston Biofabrication Institute, Metro North Hospital and Health Services, Brisbane, QLD, Australia; ^5^ Faculty of Engineering, Architecture and Information Technology, University of Queensland, Brisbane, QLD, Australia

**Keywords:** vascular, model, accuracy, 3D, quality, medical 3D printing

## Abstract

3D printing enables the rapid manufacture of patient-specific anatomical models that substantially improve patient consultation and offer unprecedented opportunities for surgical planning and training. However, the multistep preparation process may inadvertently lead to inaccurate anatomical representations which may impact clinical decision making detrimentally. Here, we investigated the dimensional accuracy of patient-specific vascular anatomical models manufactured *via* digital anatomical segmentation and Fused-Deposition Modelling (FDM), Stereolithography (SLA), Selective Laser Sintering (SLS), and PolyJet 3D printing, respectively. All printing modalities reliably produced hand-held patient-specific models of high quality. Quantitative assessment revealed an overall dimensional error of 0.20 ± 3.23%, 0.53 ± 3.16%, −0.11 ± 2.81% and −0.72 ± 2.72% for FDM, SLA, PolyJet and SLS printed models, respectively, compared to unmodified Computed Tomography Angiograms (CTAs) data. Comparison of digital 3D models to CTA data revealed an average relative dimensional error of −0.83 ± 2.13% resulting from digital anatomical segmentation and processing. Therefore, dimensional error resulting from the print modality alone were 0.76 ± 2.88%, + 0.90 ± 2.26%, + 1.62 ± 2.20% and +0.88 ± 1.97%, for FDM, SLA, PolyJet and SLS printed models, respectively. Impact on absolute measurements of feature size were minimal and assessment of relative error showed a propensity for models to be marginally underestimated. This study revealed a high level of dimensional accuracy of 3D-printed patient-specific vascular anatomical models, suggesting they meet the requirements to be used as medical devices for clinical applications.

## Introduction

Medical 3D printing (M3DP) is an emerging technology that refers to the fabrication of anatomical structures from volumetric datasets such as computed tomography (CT) or magnetic resonance imaging (MRI) images as hand-held models of patient anatomy and pathology (1). Clinical applications of M3DP range from advanced visualisation of anatomical structures and pathologies for diagnostic purposes, to enhancing patient education and physician-patient communication, research, design and testing of patient-specific implants and surgical guides, as well as providing realistic, patient-matched models for advanced surgical planning, simulation, and training (2–7). While surgical procedural planning has benefitted immensely from the increased fidelity of radiographic imaging and virtual 3D reconstructions, M3DP allows previously unattained true-to-patient tactile feedback to assist in key pre-operative planning, including patient-specific pre-operative surgical simulation (8). Literature has shown that M3DP resulted in changes to selection of endoluminal devices, reduced intraoperative contrast use and increased surgeon confidence in the field of vascular and endovascular surgery (9–11). M3DP appears particularly useful in cases of complex aneurysms where atherosclerotic disease may make vessel measurement inaccurate for selection of endoluminal devices (9, 11). Indeed, a growing body of evidence suggests that M3DP significantly improves healthcare effectiveness by reducing operating times, costs, risk of peri-operative complications, and enhancing surgical accuracy (9, 12–14). With robust systematic evidence becoming increasingly available, M3DP has gained significant momentum with healthcare providers globally establishing in-house capabilities for “point-of-care” manufacturing of patient-specific anatomical models.

The dimensional accuracy of M3DP anatomical models is crucial for physicians to correctly interpret complex spatial relationships and enable effective, safe, and evidence-driven decision-making. Inaccurate representation of anatomical features bears an inherent risk of misinformation and detrimental impact on clinical decision-making and ultimately patient outcomes. Consequently, 3D-printed patient-specific anatomical models are increasingly recognised and regulated as Class II medical devices in Australia, Europe and the United States, and are thus subject to stringent quality assurance requirements (15–19). Yet, given the comparatively recent emergence of the field, no globally recognized standard for clinically acceptable dimensional accuracy exists to-date. A small number of previous studies investigated the role of volumetric imaging parameters such as contrast and resolution, as well as anatomical segmentation procedures, tessellation density, 3D printing, and post-processing on the dimensional accuracy of 3D printed models. However, these were performed on a limited number of samples and range of anatomical feature sizes, and statistically relevant quantitative datasets assessing 3D printing accuracy remain sparse (20–23).

During the modelling and printing process there are various sources of error that may accumulate within the final printed model (21). CT and MRI are the most common sources of 3D volumetric data for anatomical reconstruction, image quality is dependent on scan settings such as slice thickness and reconstruction Kernel as well as protocols for contrast injection and timing of contrast phases (21). Segmentation is typically overseen by radiologists or personnel with specialist knowledge of desired anatomy, while there are automated and semiautomated processes these are currently not proficient enough to replace elements of manual segmentation. The segmented anatomy is converted to STL files whose geometry is optimised by modelling software to repair and smooth surfaces which may inadvertently alter key geometries (21). Accuracy of models may further be impacted by the method of printing, printer maintenance and settings to achieve optimum printing thickness and material curing. Additionally, elements of post processing may alter printed models by damaging components when removing supports and excess printing material or inadequate curing processes, however these are unique to specific printing modalities (21).

In this study, we statistically assessed the dimensional accuracy of M3DP vascular anatomical models of wide-ranging size and complexity compared to unmodified clinical imaging datasets and segmented digital anatomical models. We demonstrate that common printing modalities are well-suited for the generation of high-fidelity patient-specific models and delineate recommendations for the establishment of quality management systems in point-of-care M3DP. We further demonstrate that dimensional measurements using callipers offers a fast, inexpensive, and accurate day-to-day QA procedure for M3DP anatomical models which may be complemented by CT-scanning and congruency analysis of printed models vs. original digital segmentation to provide a complete and readily applied QA protocol assessing printing accuracy.

## Technology primer

**Figure 1:**
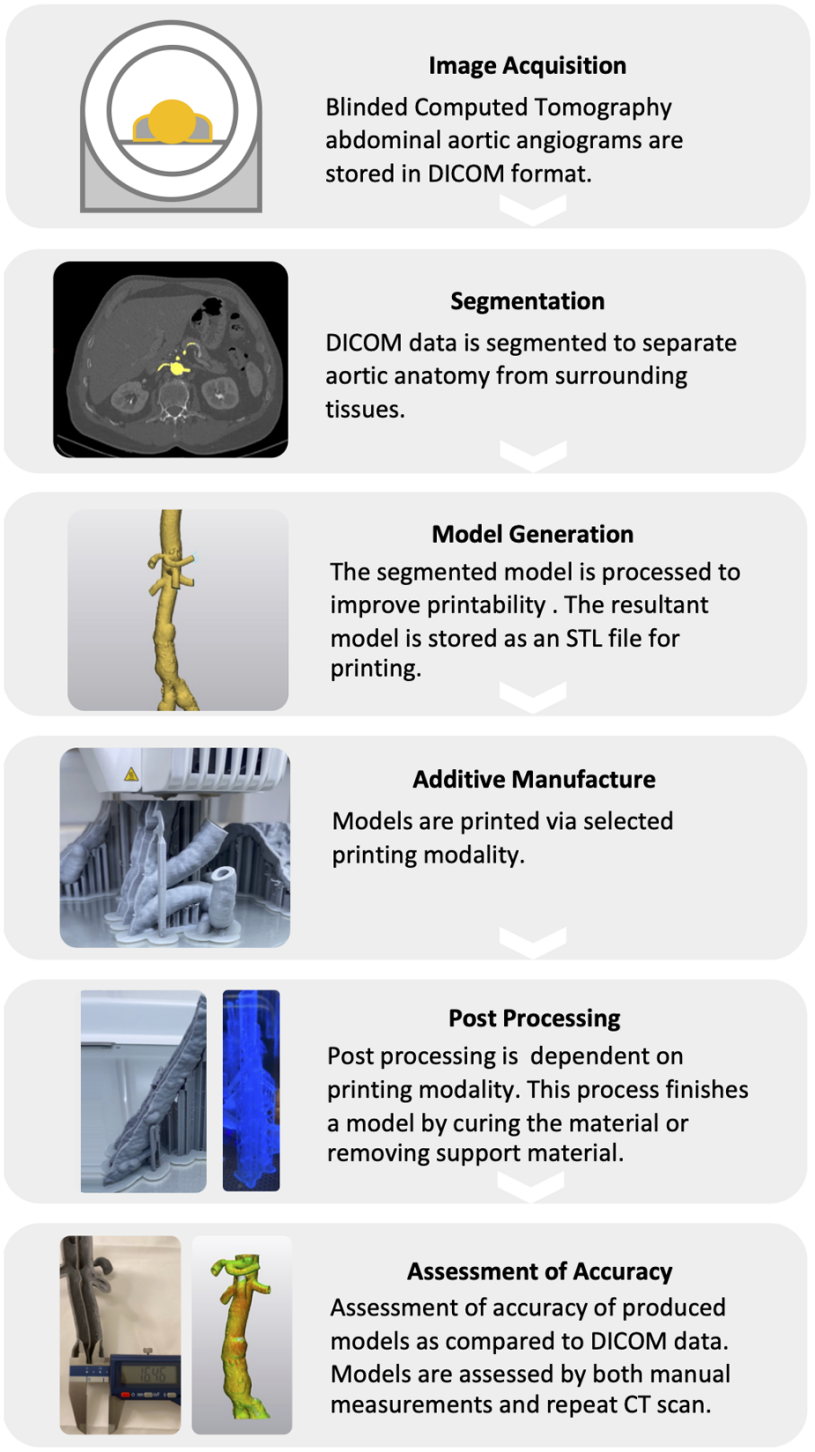
**Schematic workflow for the medical 3D-printing of vascular anatomical models.** The M3DP of anatomical models encompasses a multi-step procedure including image acquisition, digital anatomical segmentation, mesh refinement, 3D printing, and post-processing.

The M3DP process typically involves a multi-step procedure including image acquistion, anatomical segmentation, mesh refinement, 3D-printing, and post-processing of the printed modes (Figure 1).

### Image acquisition

M3DP of vascular anatomical models begins with the acquisition of 3D volumetric imaging datasets of the target anatomy with sufficient signal intensity and contrast, and minimal artefacts, to enable effective differentiation from surrounding tissues. Most commonly, M3DP models are generated from Computed Tomography (CT), CT Angiograms (CTA), or Magnetic Resonance Imaging (MRI) images, but have also been products from rotational digital subtraction angiography or 3D rotational angiography (1). The quality and accuracy of the M3DP model is inherently influenced by the quality of the imaging source data and therefore, where possible, image acquisition should incorporate electrocardiography gating, breath-holding, and MRI respiratory gating to avoid artefacts associated with cardiac movement and breathing (24).

### Segmentation

Digital segmentation is the process of identifying boundaries and areas of interest within imaging data sets which are indicative of anatomical structures of interest. This process is typically performed manually or semiautomated within segmentation software such as Materialise Mimics and relies on differing pixel attenuations within imaging datasets resultant from the varying densities and cellular structures of organs in the body (1, 25). Manual segmentation is more accurate than fully automated processes relying on interpretation by trained personnel requiring greater time input (26). Fully automated techniques are continually being improved and typically involve clustering processes and anatomical atlas' where development of accurate automated segmentation processes varies between anatomical regions and surgical specialities (26). Current practice is often a mixture of both, beginning with semiautomated segmentation followed by manual correction (1).

### Mesh refinement

Following segmentation, the resultant 3D model or 3D mesh requires optimisation prior to 3D printing. Mesh refinement uses software to correct errors resulting from semi-automated segmentation techniques which may include extraneous or incomplete model surfaces. Stepping errors produced by interpolation of individual slices of volumetric imaging data sets into 3D environments can be lessened by smoothing model surfaces. The degree of smoothing required varies with slice thickness and slice spacing of DICOM data resulting in increasing inaccuracy where larger spaces are required to be interpolated into the 3D environment (22).

### 3D printing

**Figure 2:**
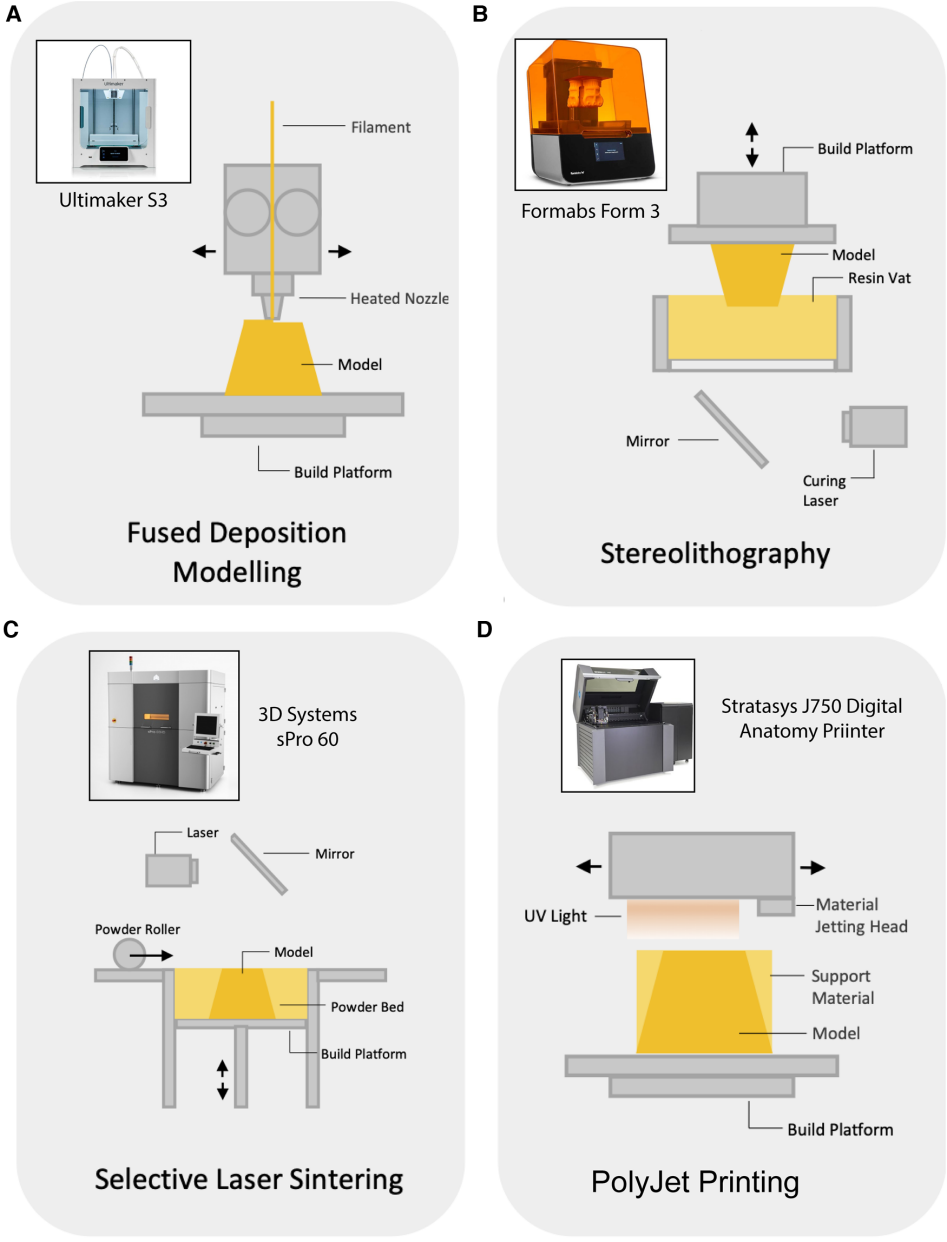
**Schematic representation of tested 3D printing modalities.** (**A**) Fused Deposition Modelling (FDM) – Common and least expensive, FDM printers heat and extrude a filament through a printhead on to a bed that descends with each layer (27). (**B**) Stereolithography (SLA) – A vat of photopolymer resin sits under a downfacing print plate which rests on the surface of the resin, the printer then uses UV laser to cure and harden a layer of the resin which is then lifted out of the resin vat in preparation for the next layer (27). (**C**) Selective Laser Sintering (SLS) – Thin layers of metal, ceramic, or polymeric powder are laser sintered to bind them together, a new layer of powder is applied and the process is repeated (27). (**D**) PolyJet printing – Print head projects particles of liquid photopolymer which is cured with ultraviolet light cured in layers and is able to produce multiple colours and materials within a single model (27).

3D printing is an additive manufacturing technology that enables the constructions of physical objects from a digital model. Over the past 4 decades a plethora of 3D printing modalities have been developed, those assessed here are described in Figure 2.

### Post-processing

Post processing refers to processing of printed models to remove support material required to stabilise the model during printing, or to cure materials to achieve desired material strength and finish. Often, FDM models are printed with support material to provide printing surface for sections of overhang, these are easily removable and are joined with a weaker bond or designated support material that is easier to remove (28). SLA resin models are washed in isopropyl alcohol to remove uncured resin followed by UV light curing to set printed resin (28). SLS Models are printed in a powder container requiring brushing and sandblasting to remove excess powder (28). PolyJet support material can be broken away manually followed by caustic solution wash to dissolve remaining or inaccessible support material (28).

## Materials and methods

### Digital anatomical segmentation

Volumetric imaging data acquired for this study comprised 11 blinded high-resolution contrast-enhanced CT abdominal aortic angiograms with 1 mm sections of normal and abdominal aortic aneurysm (AAA) anatomy, as well as femoral arteries. DICOM data was imported into Materialise Mimics (Version 24) for segmentation of vascular anatomy using the semi-automated “threshold” and “region grow” tools to delineate vascular blood volume. Where required, segmentation was refined using the “multiple slice edit” tool. The resulting anatomy was manually trimmed of extraneous vessels by editing the current mask and exported as a stereolithographic file (STL).

### Digital model generation

The STL file containing the desired segment of vascular anatomy was imported in Materialise 3matic (version 24). Here, the model was re-meshed and the “hollow” function was applied to create a digital model of the vessel wall (2.5 mm thickness (29, 30)) based on internal blood volume derived from patient CTA scans. Measurement reference points (small arrow-shaped extrusions) were then added throughout the vascular model to aid in physical assessment of resultant printed models compared to the unmodified DICOM datasets and the digital model as outlined below (Supplementary Figure S1). Additionally, all models irrespective of whether pathological or healthy were digitally dissected through the lumen centrelines to create a second sample set that allowed for measurement of internal dimensions such as lumen diameter.

### 3D printing and post-processing

Dissected and non-dissected digital models were imported as STL files into each printing modality's proprietary slicing software and supports were generated automatically following manufacturer recommendations. Anatomical models were 3D-printed using common materials and standard, modality-specific settings, and post-processed as advised by the manufacturer (Table 1).

**Table 1:** 3d printing modalities and parameters.

Printer Model	Type	Material	Support material	Layer thickness	Postprocessing
Ultimaker S3	FDM	Polylactic acid (PLA)	Polylactic acid (PLA)	0.10 mm	Manual removal of supports with pliers
Formlabs Form 3	SLA	Formlabs Grey Resin	Formlabs Grey Resin	0.05 mm	Wash in isopropyl alcohol for 30 min in the Formlabs Form Cure, air drying, postcure in the Formlabs Form Cure for 15 min at 60 °C
3D Systems sPro 60	SLS	DuraForm® Polyamide (Nylon)	–	0.10 mm	Remove excess powder with brush/compressed air, sandblasting
Stratasys J750 Digital Anatomy	PolyJet	Vessel Wall-Compliant	SUP76 B (external) GelSupport (internal)	0.014 mm	Manual removal of support material Wash in caustic solution for 24 h

### Assessment of dimensional accuracy

Small arrow-shaped extrusions (1 × 1 × 1 mm, l × w x h) were introduced to the digital anatomical model in Materialise 3Matic in approximately 2.5–3 cm intervals at arbitrary positions to serve as measurement reference points. Following 3D printing, distances between measurement reference points were determined manually on the printed anatomical models using Dasqua^TM^ Vernier callipers. Reference measurements were performed on the digital model (STL file) in Materialise 3Matic and the unmodified DICOM dataset in Materialise Mimics.

Surface congruency analysis has been performed using representative aortic models following previously published methodology (31). Briefly, printed models underwent CT scanning and repeat processing (segmentation and mesh refinement in Materialise 3Matic) to generate digital 3D models. Both the source digital model (which acted as a blueprint for 3D printing) and digital models generated from CT-scanned prints were overlayed/aligned in Materialise 3Matic using the “interactive translate” and “global registration” tools, followed by “part comparison analysis” to assess the deviation of surfaces between the models.

### Statistical analysis

One-way analysis of variance (ANOVA) with Tukey's post-hoc tests were performed using GraphPad Prism software (version 9; GraphPad, CA, United States) with a significance level of 0.05. Statistical differences are indicated in figures using symbols.

## Results

### Direct correlation of 3D printed models to patient CT scans

**Figure 3:**
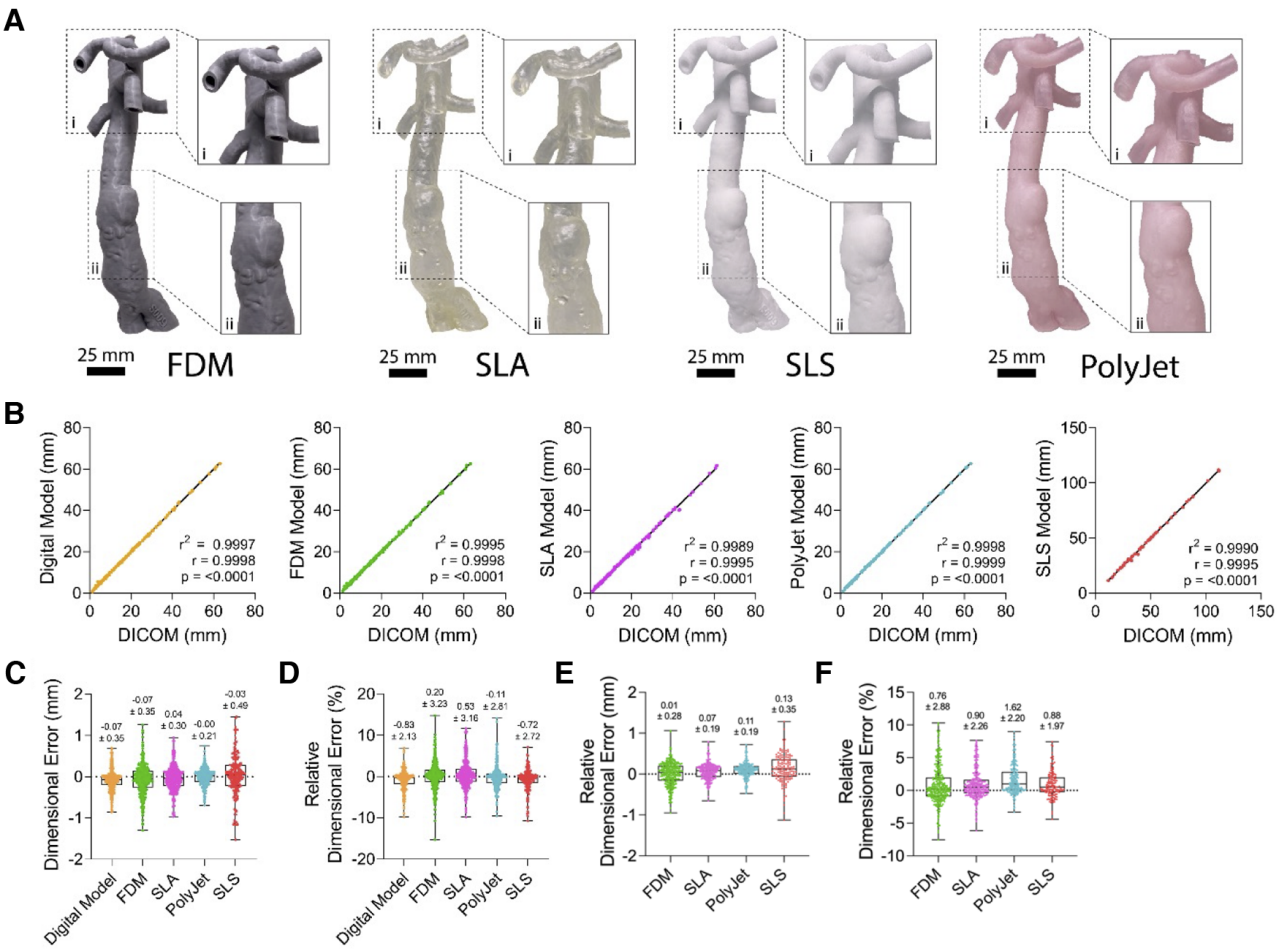
**Gross morphological appearance and dimensional accuracy of vascular M3DP models.** (**A**) Gross appearance of M3DP models printed using FDM, SLA, SLS, and POLYJET, respectively. (**B**) Dimensional correlation, (**C**) absolute and (**D**) relative error of digital and 3D-printed models compared to unmodified clinical imaging datsets (DICOM), (**E**) absolute and (**F**) relative error of 3D-printed models compared to digital models.

With the aim of validating the dimensional accuracy of vascular M3DP models, we assessed the correlation of various points of each printed vascular model with the corresponding CT scan. All printing modalities tested (FDM, SLA, SLS, and PolyJet) produced realistic hand-held models of patient anatomy (Figure 3A). Digital anatomical segmentation and mesh-refinement resulted in the creation of highly accurate digital models of patient anatomy with excellent dimensional correlation to unmodified clinical imaging dataset (*r* = 0.9998, *p* < 0.0001; Figure 3B) and negligible absolute (- 0.07 ± 0.35 mm; Figure 3C) and relative error (- 0.83 ± 2.13%; Figure 3D). All printing modalities displayed a strong positive correlation between M3DP models and CT scans (*r* > 0.9 and *p* < 0.0001 for all printing modalities) with excellent goodness of fit (*r*^2^ > 0.99 for all printing modalities; Figure 3B) and minimal average dimensional error (Figures 3C,D). Similarly, all M3DP models showed a strong positive correlation (*r* > 0.999, *r*^2^ > 0.99) with their respective digital models and minimal absolute error (0.13 ± 0.35 mm; Figure 3E) and relative error (1.62 ± 2.20%; Figure 3F). No significant differences were found between printing modalities, suggesting all tested modalities were well-suited for the creation of patient specific M3DP models. Furthermore, no differences were observed between models of normal vs. pathological anatomy.

### Effect of feature size on dimensional accuracy of M3DP models

**Figure 4:**
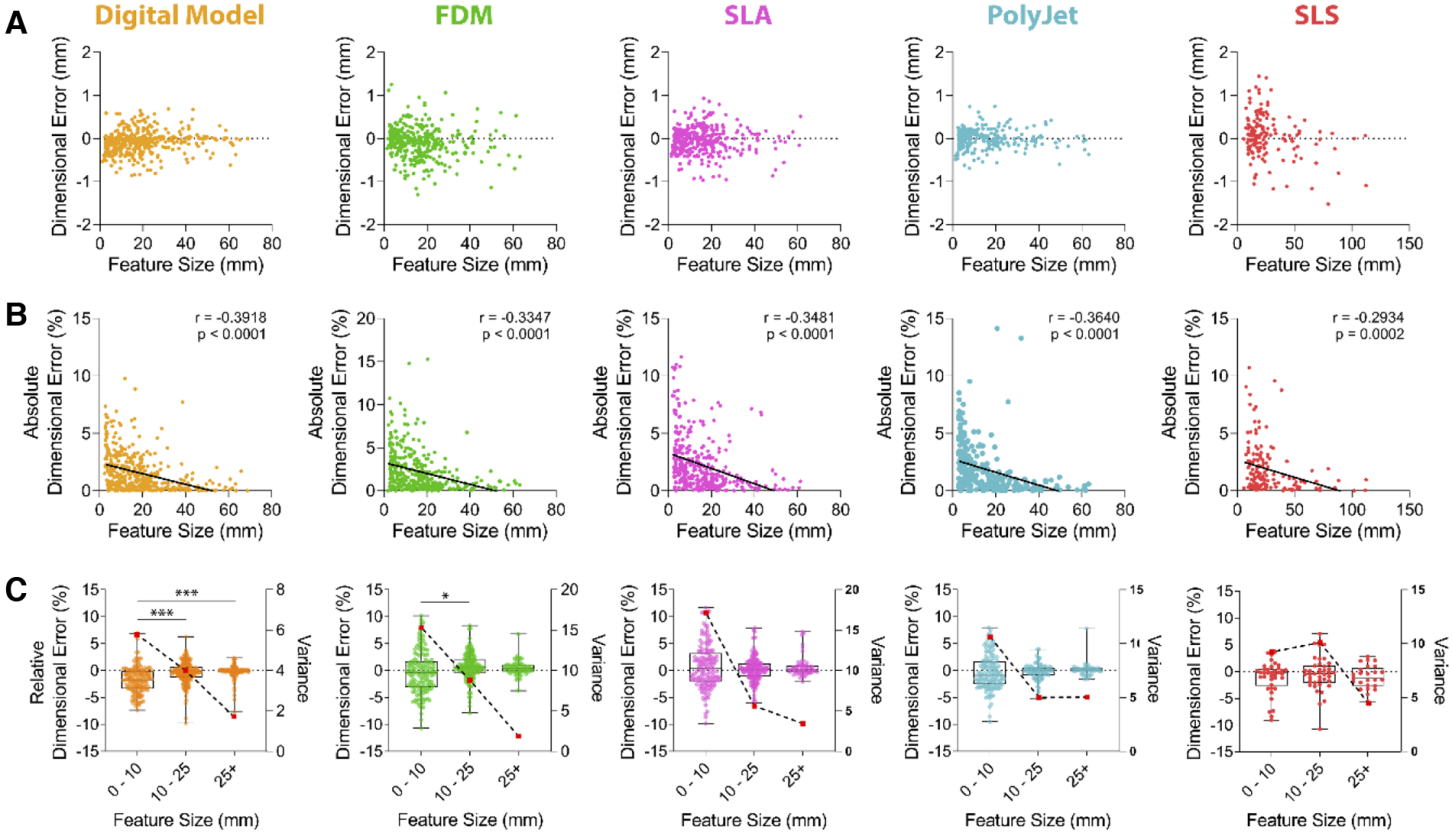
**Dimensional accuracy of vascular M3DP models as a function of feature size.** Dimensional error of digital and M3DP models as compared to original volumetric imaging datasets shown as (**A**) aimensional error in millimeters, (**B**) absolute dimensional error in percentage deviation (solid line demonstrates linear regression analysis; *r* = Pearson's correlation coefficient) and (**C**) over discreet feature size ranges, 0-10 mm, 10-25 mm and over 25 mm (relative dimensional error is shown in boxplots and plotted on left axis; Variance is indicated using broken line and red dots and plotted on right axis).

Further assessment was sought to identify if the size of an anatomical feature had an impact on dimensional accuracy when printed in each printing modality (Figure 4). Our data demonstrated a significant (*p* < 0.0001; Figure 4B) negative correlation between feature size and dimensional accuracy. Increasing feature sizes resulted in a decrease of the associated relative dimensional error across all printing modalities where there is no error between feature sizes of 41.32–66.91 mm (95% CI) and larger, except in the case of SLS (66.34–157.1 mm (95% CI); Figure 4B).

To determine if feature size influenced modelling and printing accuracy, we depicted relative dimensional error as a percentage compared to CT scan data over discreet ranges of 0–10 mm, 10–25 mm and over 25 mm (Figure 4C). Digital model generation results in statistically significant error between these ranges (Figure 4C). Variance, a measure of data dispersion, substantially decreased with increasing feature sizes across all tested printing modalities, suggesting dimensional errors are most variable when small features are printed (Figure 4C). Variance within the 0–10 mm range was highest FDM and SLA printed models. At feature sizes over 25 mm, the dimensional error was under 1% for all models (FDM 0.47 ± 1.39%, SLA 0.68 ± 1.89%, PolyJet 0.14 ± 1.30%, SLS −0.97 ± 2.15%; Figure 4C) with variance for FDM, SLA, SLS and j750 of 1.89, 3.46, 4.45, 5.05 respectively.

### Analysis of surface congruency

**Figure 5:**
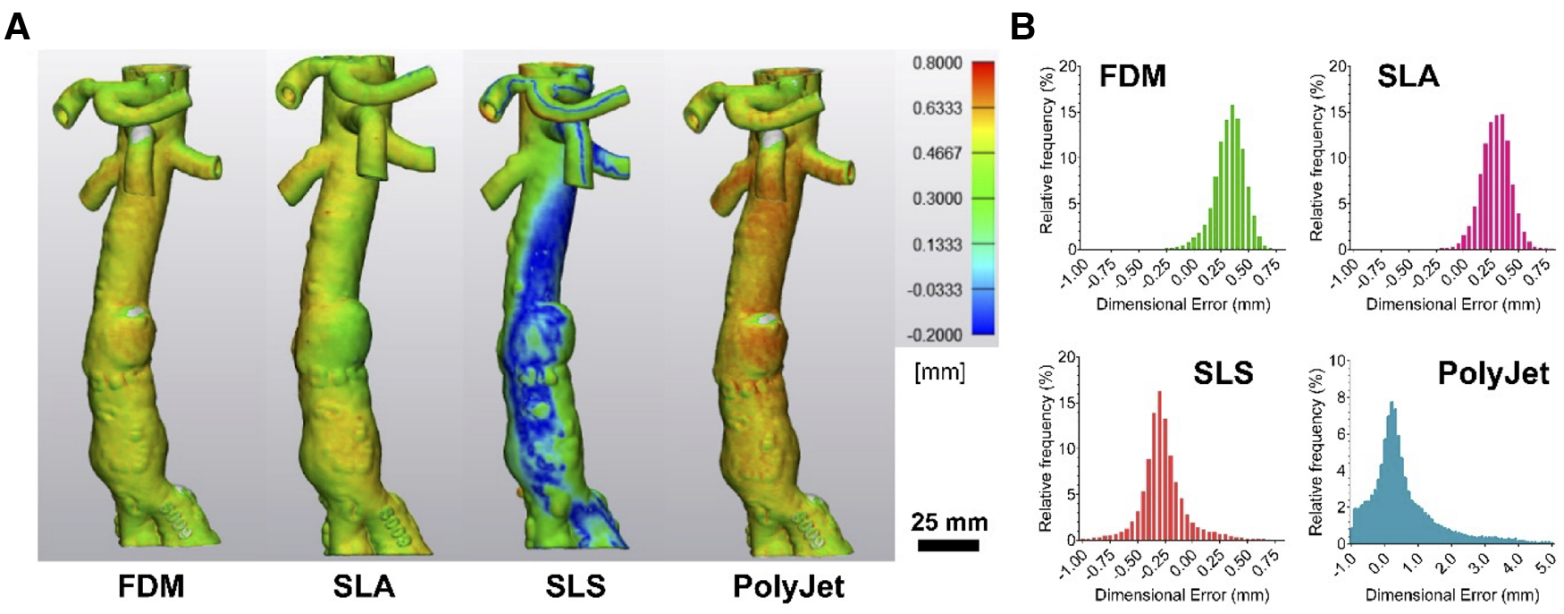
**Surface congruency analysis of 3D-printed models compared to digital model.** (**A**) Representative images of surface congruency analysis of FDM, SLA, SLS, and PolyJet printed anatomical models and (**B**) histograms demonstrating data distribution.

Further assessment of dimensional accuracy of printed 3D models was performed following a surface congruency analysis method developed by Dorweiler et al. (31) (Figure 5A). Overall, models demonstrated largely congruent surfaces with the presence of hotspot and areas of surface deviation when compared to the original digital model which served as a blueprint for the 3D printing process. Mean surface deviations were 0.322 ± 0.15 mm, 0.29 ± 0.14 mm, −0.27 ± 0.21 mm, and 0.58 ± 1.09 mm for FDM, SLA, SLS, and PolyJet printed models, respectively (Figure 5B).

**Figure 6:**
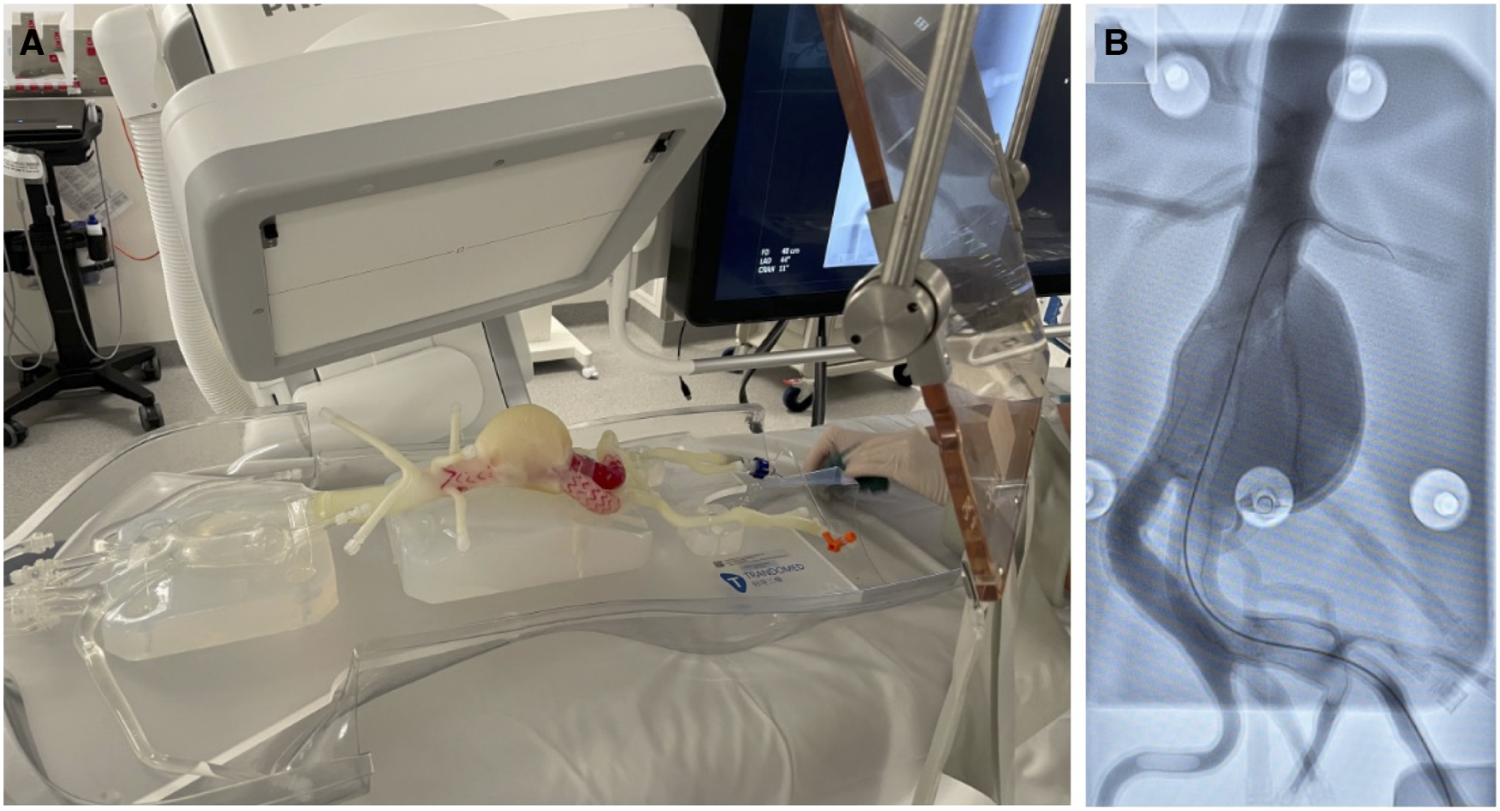
**Example application of M3DP abdominal aortic aneurism (AAA) model for surgical training.** (**A**) Perfused AAA model situated under x-Ray C-Arm and (**B**) representative x-Ray image showing contrast-enhanced model and surgical guide wire.

Using completed models, we developed a case simulation of endovascular aortic repair (EVAR) utilising a contrast filled pulsatile pressure pump to simulate systolic and diastolic blood pressure variations (Figure 6). This system was setup with x-Ray C-Arm *in situ* (Figure 6A) with representative x-Ray image of a surgeon practicing guidewire insertion into the abdominal aorta (Figure 6B). The model was printed using PolyJet technology and a tissue-mimetic elastic material that provided realistic tactile feedback and appearance on x-Ray.

### Discussion

Good quality management systems are an integral part to the manufacture of any commercial medical device and are typically governed by strict protocols to ensure quality and reproducibility (15–17). The same principles are relevant to smaller centres of additive manufacturing creating personalised medical devices or equipment. Here, we have sought to validate two methods for assessing dimensional accuracy of additive manufactured vascular models in the hopes that these methods may be adopted into future quality management systems to streamline the use of point of care 3D printing.

### Accuracy of digital model creation

We have illustrated inherent dimensional discrepancies throughout the workflow of creating patient specific vascular models. The first point for introduction of error is the patient CT scan. Here, the thickness and resolution of each layer of the scan has a flow-on effect to the segmentation process whereby desired anatomy is segmented by functions of 3D modelling software and which is often completed by hand (21, 26). Segmentation completed automatically by modelling software, or in this case Materialise Mimics, and relies on brightness and contrast of pixels of the scan to delineate structures which may be affected by changes in the concentration of contrast or post processing of the scan (25, 32). Slice thickness and the space between slices determines how much data that modelling software needs to interpolate in order to generate a 3D model, and thus larger gaps between slices or thinner slices reduces the accuracy of models produced (22). Often anatomical areas of interest are segmented by operators and thus an element of human error exists, this is typically minimised with segmentation ideally performed by radiologists trained in CT (21, 25). For the purposes of this study segmentation was performed by the primary investigator with correlation from interpretation of abdominal CT scans. While there exists small deviations due to factors discussed above, the overall impact across feature sizes, provided accurate segmentation has occurred is negligible.

### Accuracy of different printing modalities

We have found the use of Vernier callipers in assessment of vascular anatomical models has a strong correlation to corresponding components as measured from CT data (Figure 3B). The use of callipers as a means of quality control has been validated across orthopaedic and dental fields and is simple, as well as cost- and time-effective (33–35). George et al. suggested that cardiovascular M3DP models are comparable to their orthopaedic counterparts but have more inaccuracy due to the nature of soft tissue (36). The addition of markers for measuring aims to reduce measurement error by reproducibly locating landmarks (22). As such we have provided further evidence that vernier callipers are accurate, cheaper alternative to other methods such as CT scanning printed models for assessment of vascular models.

Assessment of dimensional accuracy of models printed by FDM, SLS, SLA and PolyJet printers supports these modalities as dimensionally accurate to produce large calibre vascular models. Indeed, where similar studies of aortic models show PolyJet printers to have a mean surface deviation of 0.15 mm as suitable for M3DP we have shown accuracy of all tested modalities well exceeds this at under 0.07 mm deviation (Figure 4C) (31, 36, 37). The dimensional error (Figures 4B,C) at smaller feature sizes suggests these printing modalities may even be sufficient for microvascular M3DP of small arteries typically 100–400 µm, such as cerebral or coronary vasculature; however, this has not been validated here (38). Models created for the surgical planning or procedural practice that involve systems of small vessels may therefore not be suitable for these purposes however in a physiological context the natural elasticity of vessel walls may alleviate the necessity for extremely accurate hard models. Overall, the accuracy of models produced increases dramatically as the size of the feature increases (Figure 4C), with variance approaching zero as feature sizes exceed 25 mm.

On an individual basis, all four printing modalities have been found to be accurate with overall dimensional error well below 1 mm and thus suitable for the creation of aortic vascular models (Figures 3C,D). As mentioned above, the creation of digital models results in an average error of −0.83 ± 2.13%, resultant from segmentation and digital model optimisation. Additionally, we compared the accuracy of printed models to their digital model counterparts to assess accuracy of manufacturing workflows (Supplementary Figure S2). All printing modalities displayed a high degree of dimensional accuracy with average dimensional errors of printing for each modality being less than 1 mm (Figure 3E).

All models across the four printing modalities tested have shown submillimeter accuracy in surface congruency analysis (Figure 5) in line with suggested accuracy for M3DP (30, 36). Differences in FDM, SLA and SLS model accuracy as compared to calliper measurements may be the result of printing specific processes. FDM models are susceptible to deformation during cooling and post processing particularly without judicious use of support material and optimal positioning. Support material in FDM and SLA models may lead to surface incongruency where smaller vessels such as superior mesenteric, coeliac trunk and renal arteries have internal supports required for printing that cannot be removed and are then detected as internal structures during CT assessment (31, 36). Whole SLS model underestimation was unexpected and can be postulated to be the result of model shrinkage or loss of finer details to the porous finish of SLS models, indeed we found that many of our reference markers were lost to post processing (39).

### Elastic models

Each printing modality is significantly different from the next. Changes in production technique, material, and environment all impact on the quality and characteristics of the final device. Here, we have printed primarily with hard finish polymeric materials to enable measuring of different features; however, in certain scenarios a soft model may be preferable due to its ability to mimic the elasticity of patient blood vessels as well as internal atherosclerotic plaques (9). Soft models therefore have increased use in scenarios where the physics of a vessel may be important such as endovascular procedural training using vascular models in a circuit of liquid under pulsatile pressure (Figure 6) or selection and design of standard and fenestrated endovascular grafts (6, 9–11, 40) While elasticity in these models aims to mimic human anatomy, it is inherently difficulty to interpret and replicate the complex strains put across vessel walls especially in the presence of patient specific pathology such as dissections or aneurysms (11).

### Limitations of printing modalities in a point of care setting

Assessment of literature in both orthopaedic and vascular spaces reveals a gap in the validation of multiple printing modalities for use in vascular anatomical modelling (9, 36). When selecting a printing modality for M3DP, some modalities lend themselves to more practicable small volume construction. Most FDM and SLA printers are purpose built for small volume manufacturing and lend themselves to prototyping which is ideal for patient specific point of care devices; however, have long printing times (41) SLS printers are less suited to this purpose as they require full powder bed and machinery set up regardless of the size or quantity of printing (41). PolyJet printers are the most versatile method of manufacture, its print bed allows the manufacture of multiple models in different materials and is not subject to lengthy set up processes. Additionally, PolyJet printed models have dissolvable gel support material minimising post processing and handling which may further impact model accuracy. Preparation and production time remain a limiting factor of M3DP in emergency surgery with printer preparation taking from 20 min to 48 h and production at 9 h to 16 days (9).

Cost varies significantly between printers which has previously been described by Serran et al*.*, who break down ongoing costs into materials, staff costs, maintenance of printer, electricity and ancillary services which all vary based on printing modality (42).

## Conclusion

This study demonstrates that FDM, SLA, SLS and PolyJet printers are able to accurately produce patient-specific 3D models of aortic vascular anatomy with less than ± 2% overall dimensional error. It further demonstrates a significant negative correlation between the anatomical feature size printed and the resulting accuracy, suggesting particular care must be taken to ensure small anatomical features are printed sufficiently accurate. Our results further demonstrate the suitability of simple QA procedures utilising calliper measurements of reference points for clinical application of M3DP.

M3DP anatomical models have the potential to improve patient health outcomes by improving procedural planning and developing models for development of, and training in endovascular procedures and device placement. The workflows required to generate these models should be governed by Quality Management Systems to comply with ISO 13485 and we have shown that 3D model assessment with Vernier callipers is comparable to CT scanning and digital comparison of 3D printed models to original CT scans for quality assurance purposes.

Quantitative assessment revealed an overall dimensional error of 0.20 ± 3.23%, 0.53 ± 3.16%, −0.11 ± 2.81% and −0.72 ± 2.72% for FDM, SLA, PolyJet and SLS printed models, respectively, compared to unmodified Computed Tomography Angiograms (CTAs) data. Comparison of digital 3D models to CTA data revealed an average relative dimensional error of −0.83 ± 2.13% resulting from digital anatomical segmentation and processing. Therefore, dimensional error resulting from the print modality alone were 0.76 ± 2.88%, + 0.90 ± 2.26%, + 1.62 ± 2.20% and +0.88 ± 1.97%, for FDM, SLA, PolyJet and SLS.

## Data Availability

The original contributions presented in the study are included in the article/**Supplementary Material**, further inquiries can be directed to the corresponding author/s.
