# Supplementary figures and images for: Quality assurance in 3D-printing: A dimensional accuracy study of patient-specific 3D-printed vascular anatomical models
Source: Front Med Technol. 2023 Feb 7;5:1097850. doi: 10.3389/fmedt.2023.1097850 (PMC9941637; doi:10.3389/fmedt.2023.1097850)

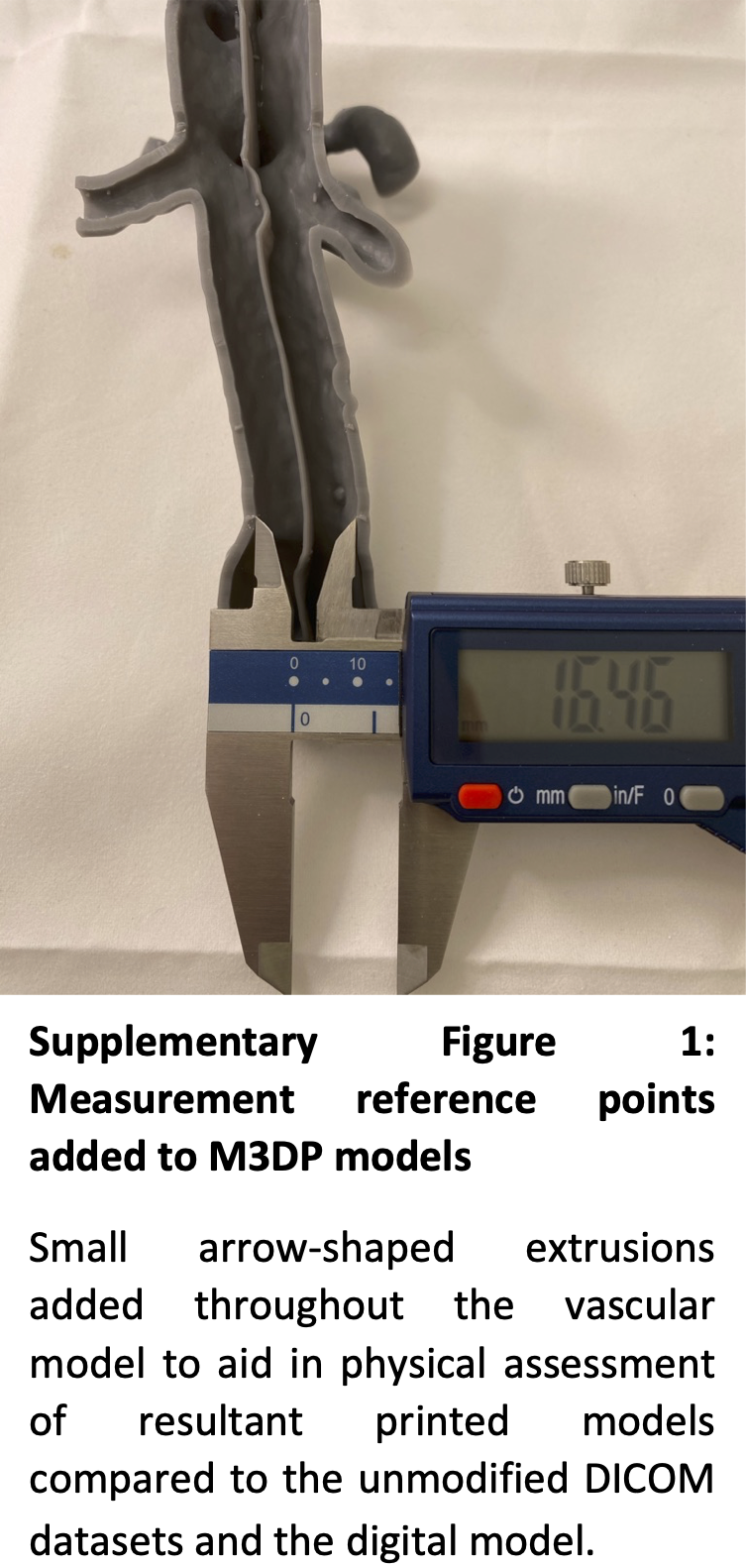

Supplement: Supplementary file 1 [file Image1.tiff]

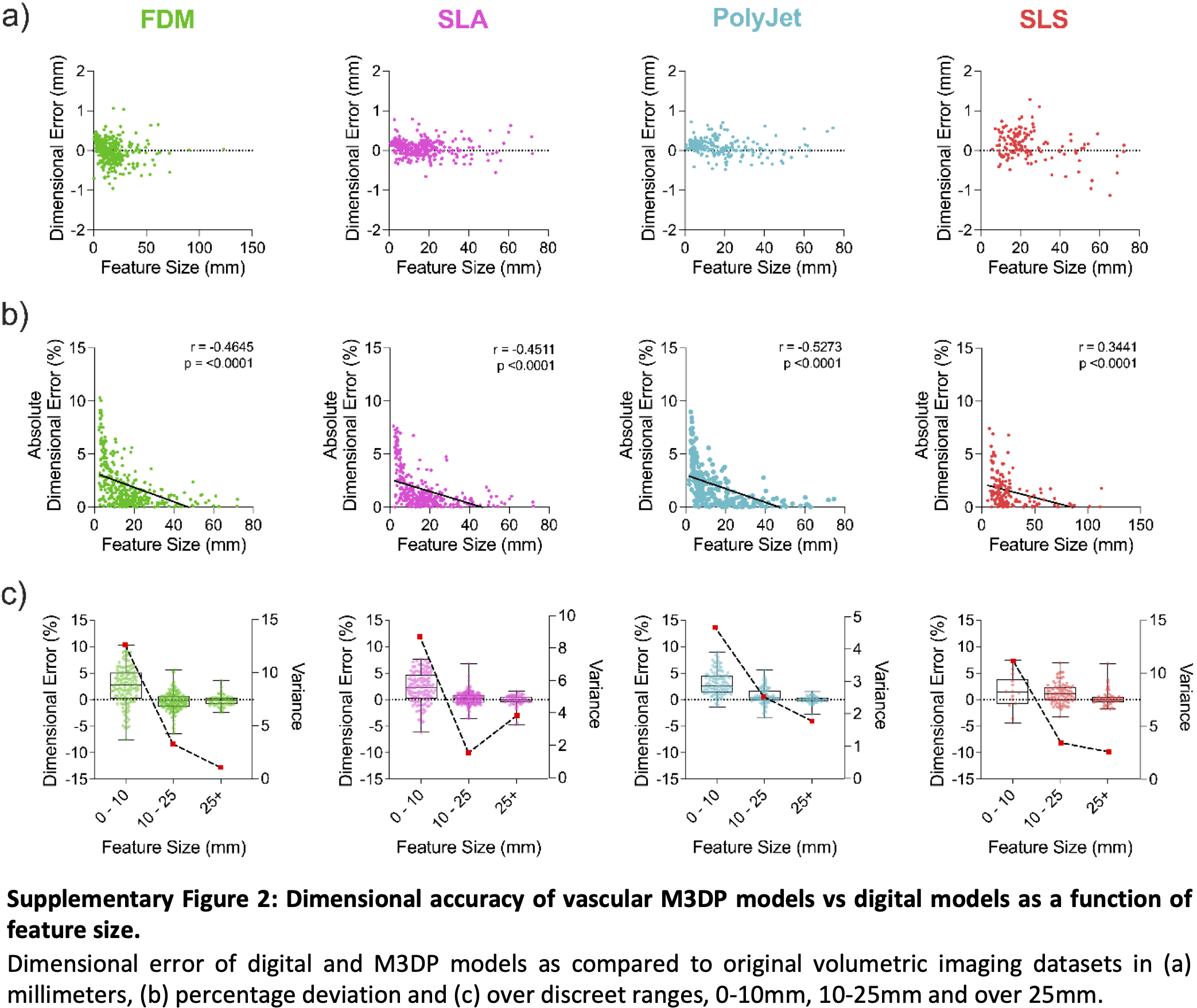

Supplement: Supplementary file 2 [file Image2.tiff]
